# The Evolution of Functionally Redundant Species; Evidence from Beetles

**DOI:** 10.1371/journal.pone.0137974

**Published:** 2015-10-08

**Authors:** Marten Scheffer, Remi Vergnon, Egbert H. van Nes, Jan G. M. Cuppen, Edwin T. H. M. Peeters, Remko Leijs, Anders N. Nilsson

**Affiliations:** 1 Department of Aquatic Ecology and Water Quality Management, Wageningen University, P.O. Box 47, NL–6700 AA, Wageningen, The Netherlands; 2 South Australian Museum, North Terrace, Adelaide, South Australia, 5000, Australia; 3 School of Earth and Environmental Science, University of Adelaide, Adelaide, South Australia, Australia; 4 School of Biological Sciences, Flinders University of South Australia, Adelaide, South Australia, Australia; 5 Department of Ecology and Environmental Science, University of Umeå, S–901 87, Umeå, Sweden; University of Melbourne, AUSTRALIA

## Abstract

While species fulfill many different roles in ecosystems, it has been suggested that numerous species might actually share the same function in a near neutral way. So-far, however, it is unclear whether such functional redundancy really exists. We scrutinize this question using extensive data on the world’s 4168 species of diving beetles. We show that across the globe these animals have evolved towards a small number of regularly-spaced body sizes, and that locally co-existing species are either very similar in size or differ by at least 35%. Surprisingly, intermediate size differences (10–20%) are rare. As body-size strongly reflects functional aspects such as the food that these generalist predators can eat, these beetles thus form relatively distinct groups of functional look-a-likes. The striking global regularity of these patterns support the idea that a self-organizing process drives such species-rich groups to self-organize evolutionary into clusters where functional redundancy ensures resilience through an insurance effect.

## Introduction

**Fig 1:**
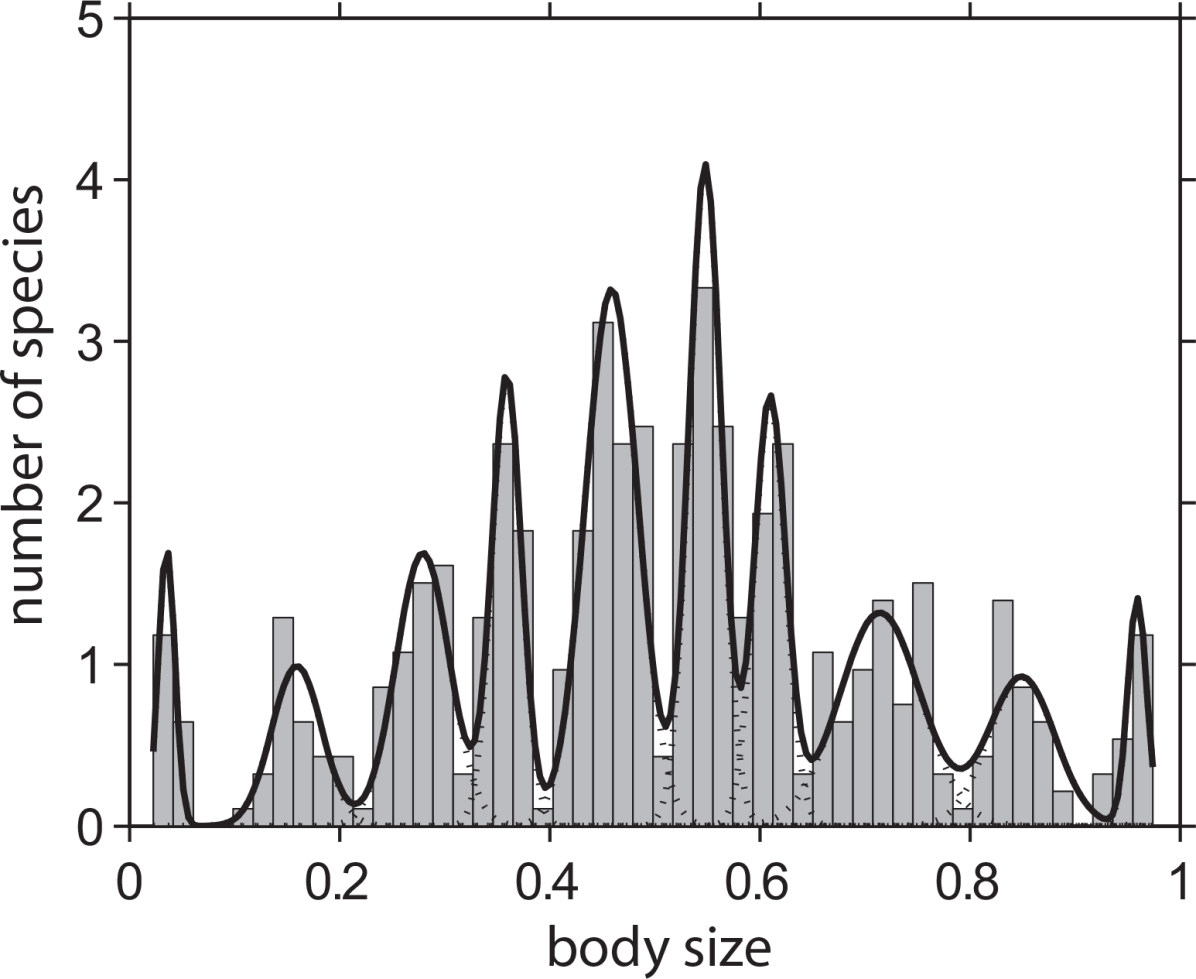
Example of a self-organized size distribution of species predicted by a generic model of competition and evolution along a hypothetical niche axis [13]. Evolutionary self-organization leads to lumps of similar species, where the spacing between the lumps corresponds to classical limiting similarity. The curves represent Gaussian distributions fitted through latent class analysis using the ‘gmdistribution’ function from the MATLAB statistics toolbox (MATLAB version R2011a) to find the best fit for models with 1–10 classes. See S1 File for model and S2 File for mode analysis.

‘Why are there so many kinds of animals?’ Hutchinson asked half a century ago in what became a classic article in ecology [1]. As he pointed out, it is hard to imagine that there are really so many different niches. Follow-up work showed that competitive exclusion through the ‘limiting similarity’ principle [2] may in practice be prevented by forces ranging from predation [3] to chaotic population dynamics [4], environmental fluctuation [5,6] and incidental disturbances [7,8]. Radically different is the idea proposed in the neutral theory of biodiversity [9,10], suggesting that large numbers of species may co-exist simply because they are equivalent, so that no species can out-compete another. This theoretical result is fragile in the sense that if one lets species differ slightly, the co-existence collapses [11,12]. However, recent theoretical work has suggested a way out of the paradox, proving that the neutrality idea can be merged with niche theory, to produce predictions of co-existence of many species in a limited number of niches [13,14]. The central result is that a particular niche may fill up with a large number of species which are not completely identical, but almost equivalent. Such near-neutrality makes the process of competitive exclusion slow enough to be offset by the many equalizing mechanisms that help prevent competitive exclusion. Proving near-neutrality within a niche is hard, as there can always be unnoticed extra niche dimensions in which the species differ. However, predictions become more specific and testable if rather than a set of idiosyncratic niches there is a continuous niche dimension, such as a size range of food items. In this situation species that differ more in size are expected to compete less as they can utilize differently-sized food items. Evolution is then predicted to lead to self-organized groups of similarly-sized species, with a regular spacing between such groups (e.g. Fig 1) [13]. Mathematically, this is essentially similar to the explanations for emergent regular patterns such as the stripes on a zebra skin or the tiger-bush pattern of desert vegetation. The regularity is what sets such patterns aside, and what allows us to recognize them as the result of self-organization in nature [15]. The basic principle behind such self-organization was demonstrated in a classical paper by the legendary mathematician Alan Turing [16]. Essentially, such regular patterns emerge when conditions are such that a homogeneous distribution becomes unstable. As a result of such ‘Turing instability’ even a small perturbation is enough to break the symmetry and lead to a self-amplified redistribution into regular patterns. In the case of animal skin patterns or desert vegetation such patterns occur in physical space. In the case of competition, the patterns are formed in niche space.

The robustness of the tendency for self-organization in niche-space has been demonstrated analytically and numerically in a range of competition models including more than one niche dimension [17,18]. Also, several sets of field data seem to support the predictions [15,19,20]. Nonetheless, the idea of (near) neutrality remains highly controversial and it is at the heart of the debate on the functional role of biodiversity, as it seems a paradox in view of niche theory [21]. Indeed, if self-organized similarity shaped much of nature’s diversity we should fundamentally rethink the way we look at the ecological identity of species [22,23].

## Results and Discussion

Here we confront the prediction of self-organized similarity to massive data from the most species-rich group of animals in nature: beetles. We focus on global data for adult diving beetles (Dytiscidae). Body size in these animals will reflect aspects of the functional niche, as it does in many other groups of animals [13,24]. This is because many functional aspects are related to body size. For instance, as these animals (especially their larvae) are mostly opportunistic predators [25], body size relates to the range of prey items available. The fact that body size is correlated to functional niche aspects implies that more similarly-sized animals are expected to compete more. As a first step we analyzed the size distributions of all known species of diving beetles for the different zoogeographical regions of the world as provided in the World Catalogue of Insects, Vol. 3: Dytiscidae (Coleoptera) [24]. In addition we scrutinized patterns of size difference between co-occuring species on a local scale by analyzing body length ratios of all pairs of species found in 1507 samples taken from Dutch ditches as reported in the Limnodata neerlandica (available at http://www.limnodata.nl). Lastly, we relate our findings to body size patterns in communities of subterranean species that evolved independently in isolation for 5 million years in 34 Australian aquifers [26]. To detect multiple modes in the frequency distributions of body lengths in the global data-set [24], in the simulated data and in the body size ratios of co-occurring species in the Dutch field data we fitted Gaussian distributions through latent class analysis using the ‘gmdistribution’ function from the MATLAB statistics toolbox (MATLAB version R2011a) for models with 1–10 classes (See S2 File).

### Size distributions on global and local scales

**Fig 2:**
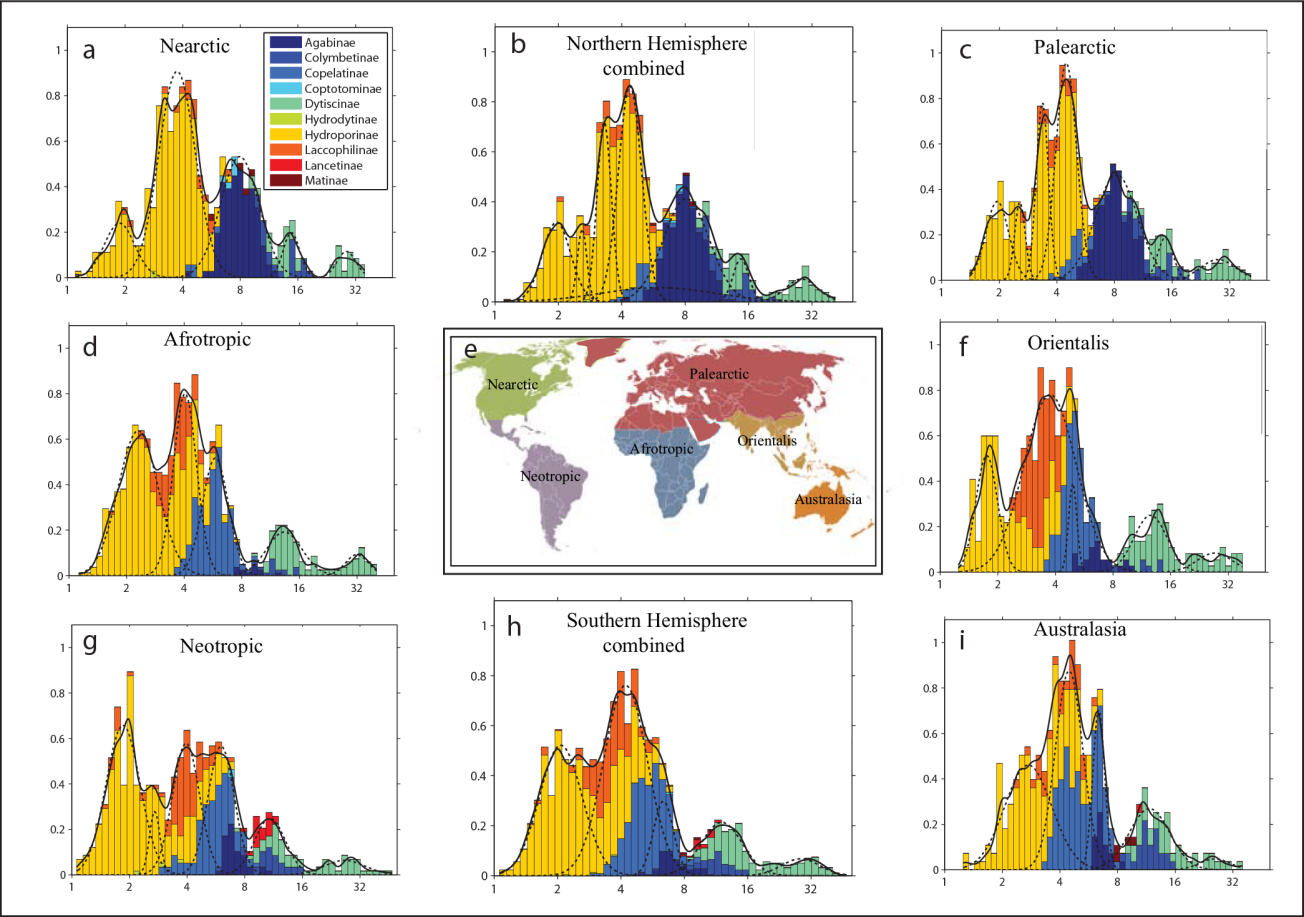
Size distributions of diving beetles in the different zoogeographical regions indicated in the central panel (e) and for the Northern (b) and Southern hemisphere (h) combined. The solid curve represents a kernel smoothed estimator of species density (see methods). Dashed curves represent the fitted constituent distributions computed by means of latent class analysis as in Fig 1. See S2 File for mode analysis.

**Fig 3:**
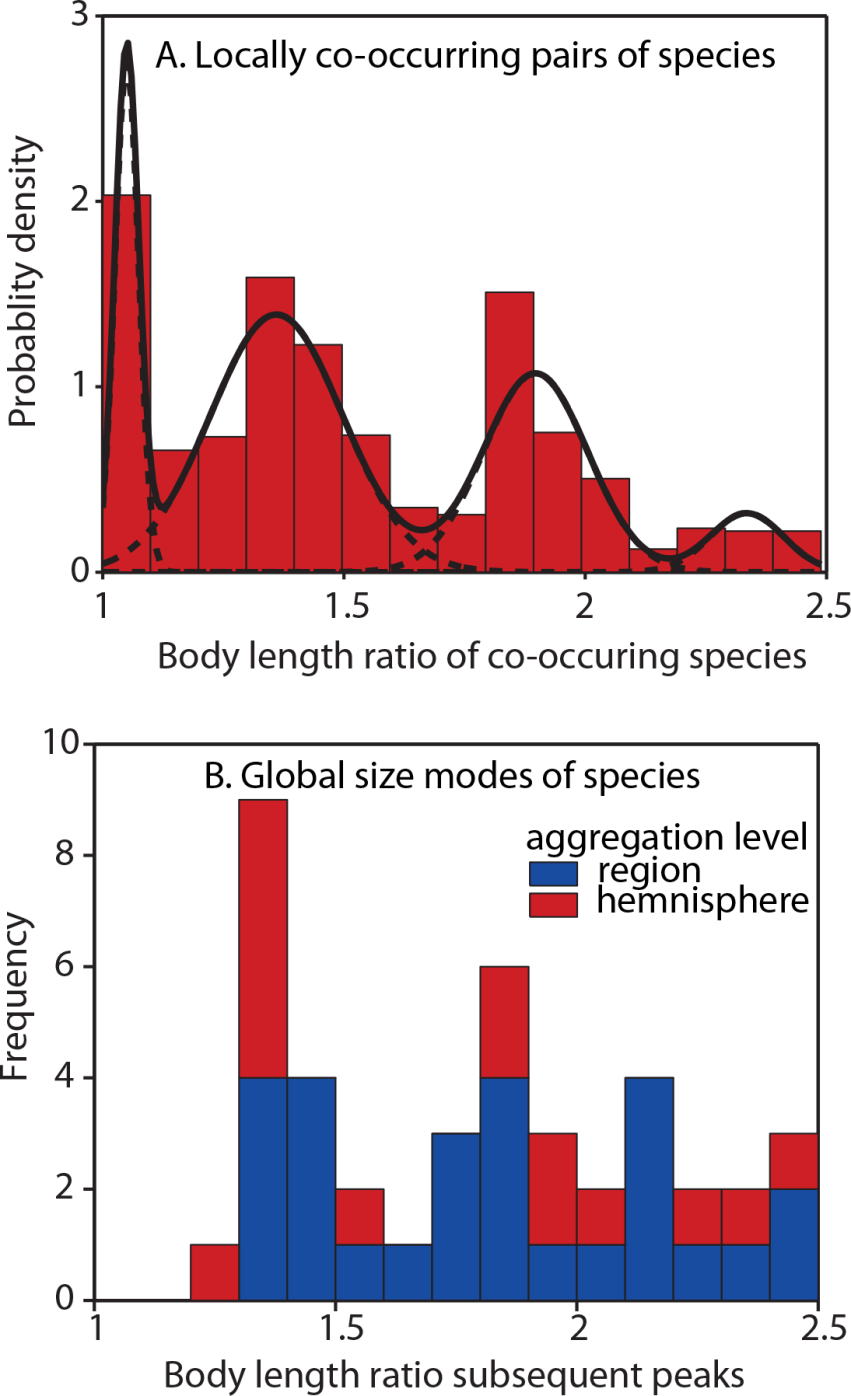
Distributions of body length ratios in diving beetles. (A) Body length ratios of pairs of species found in 1507 samples of Dutch ditches where >2 species of diving beetles were found (yielding a total of 28,762 locally co-occurring species pairs). To compute the ratios between pairs of species found in the same samples from Dutch ditches we used the average body lengths reported for those species. Subsequently we calculated the ratio of the length of all combinations of species within each sample. Dashed curves represent the fitted constituent distributions computed by means of latent class analysis as in Fig 1. (B). Frequency distribution of the peak-to-peak body length ratio for each zoogeographical region (n = 27) and of the northern and southern hemisphere aggregated (n = 11) (see Fig 2).

Our global analysis reveals that the frequency distributions of log body sizes are multimodal for all zoogeographical regions of the world (Fig 2). The regular patterns we find are consistent with self-organized niche spacing reducing competition between the modes, while at the same time allowing a large number of species to co-exist within each mode. To test if those large-scale patterns reflect true co-occurrence in the field we analyzed body length ratios of pairs of species found in 1507 samples taken from Dutch ditches (STOWA, Limnodata neerlandica at http://www.limnodata.nl). The results (Fig 3A) confirm that also on a micro-scale, co-occurring species tend to be either similar in size or differ by a factor of 1.35 (echoed by its approximate multiples of 1.8 and 2.4). This ratio is well in line with the spacing between lumps of species found across zoogeographical regions of the world (Fig 3B). The size ratio of 1.35 most likely reflects the ‘limiting similarity’ needed for niche separation. In fact, it corresponds remarkably well to the average size ratio of roughly 1.3 between co-occurring pairs of otherwise very similar species of mammals and birds discussed by Hutchinson in his classical paper [1]. Most importantly however, the micro-scale field patterns confirm that co-occurring species often have very similar sizes.

The key observation confirmed by the field observations (Fig 3A) is that while similarity and dissimilarity (size ratios of 1 versus 1.35 or more) are common, intermediate differences (size ratios around 1.1–1.2) are less likely. Given that size approximately reflects niche in these generalist carnivores, the patterns we detect on continental and micro-scales support the hypothesis [13] that evolution drives species to become either sufficiently similar or sufficiently different to co-exist. Clearly, body size is not a perfect indicator of the food range that is available to a species, as morphology of the mouth parts, habitat choice, time constraints during larval development and numerous other relevant functional aspects will vary between species. It is therefore remarkable that the 1.35 spacing pattern that stands out clearly at a micro-scale is still recognizable at continental scales.

### Repeated size evolution: a 5 million year natural replicated experiment

**Fig 4:**
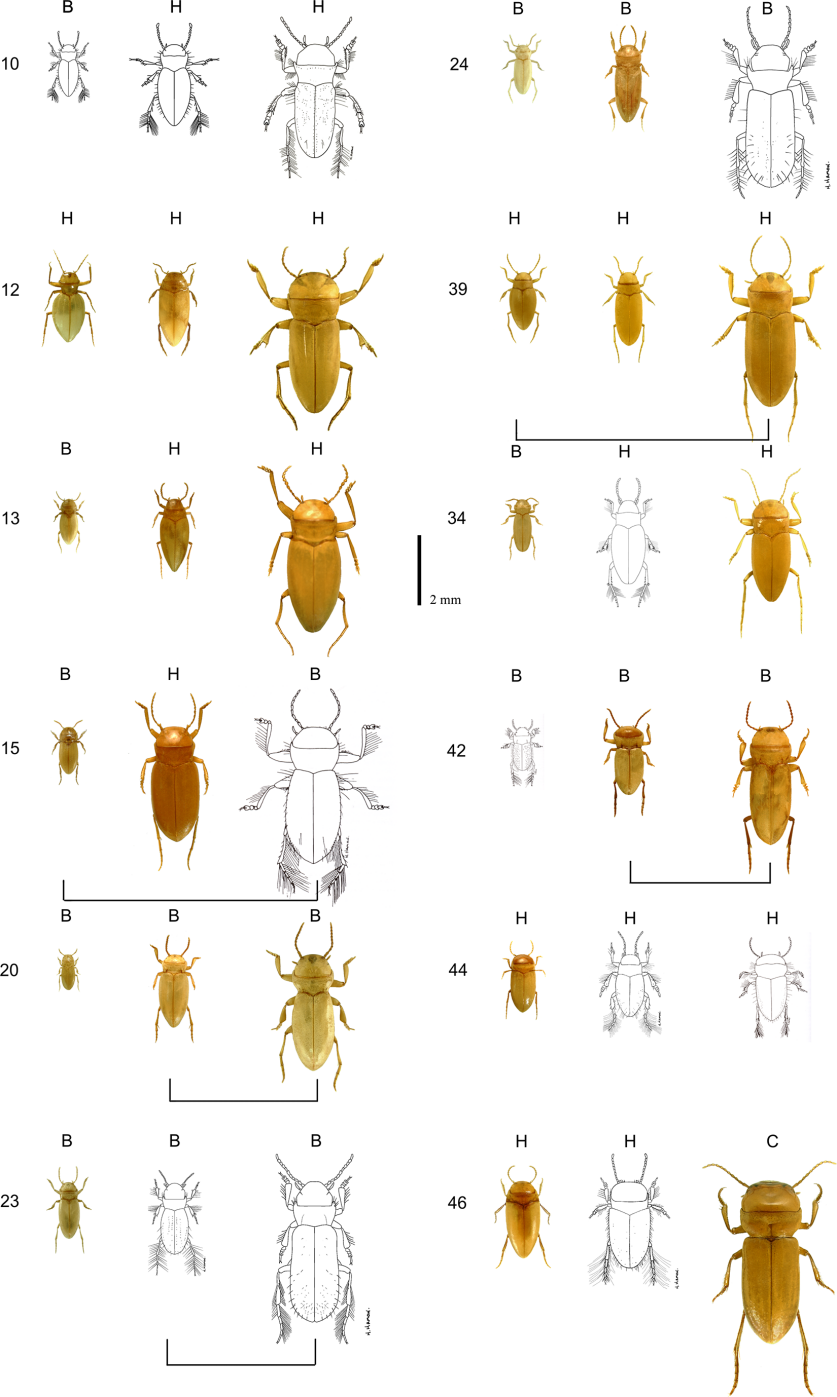
Communities consisting of triplets of blind diving beetle species found in different underground aquifers (numbers) that became isolated 5 million years ago when Australian climate became arid. Independently of the founder species, evolution led to a small a medium and a large species in each aquifer. H, B and C code the tribes Bidessini, Hydroporini and Copelatini; bars connect pairs of species that evolved from the same founder species (illustration courtesy of Chris Watts and Howard Hamond).

An obvious question is how the apparent multitude of species occupying the same niche could evolve. Sympatric convergence may seem unlikely, but is in fact expected theoretically [13] and consistent with evidence from Cichlid evolution [27]. Another possibility is that in isolated habitats adaptive radiation has repeatedly produced similar species, and that upon subsequent reconnection such communities mixed allowing near-neutral co-existence of such species. Indeed, we found a striking example of repeated self-organization in diving beetles [28,29]. In Australia around 5 million years ago climatic drying eliminated most surface water and forced diving beetles to go underground where they have since then evolved in entirely isolated aquifers. To check for size patterns, we measured species from 34 of these aquifers. Most aquifers turned out to contain three species, a small, a medium and a large one (Fig 4) [26]. Phylogenetic analysis reveals that this pattern occurs quite independently of the founder species, and that two differently sized species even evolved in several cases from a single founder-species [28]. Clearly, this is a different habitat from the open water. The maximum body size is constrained by the cavities in which the animals have to manoeuvre, and these entirely isolated habitats are poor in resources which may explain why only one species survived in each of the three self-organized niches [13,26]. Also, the size ratio between the species (roughly a factor 1.6) is larger than in the surface water communities. This larger niche spacing may seem surprising at first sight, but is in line with the prediction that species should space out more on the niche axis if there are fewer competing species [30]. Despite the idiosyncrasies this remarkable replicated natural experiment illustrates the potential for repeated evolution of similar size-structures.

### Parallel lines of theory coming together

Taken together our results support the theoretical prediction that diversity of species-rich communities may arise from evolutionary self-organization towards near-neutral co-existence of numerous species in a limited number of functional groups [13]. We do wish to stress that this finding does not come out of the blue. Rather we are standing on the shoulders of giants on both the theoretical and the empirical side.

On the theoretical side the possibility of *convergent evolution of competing species* was already predicted in 1967 by MacArthur and Levins in their classical paper on similarity [2], although it was thought at that time that upon convergence, only one species would eventually prevail in each niche. The insight that in a complex world with numerous competing species such forces could lead to the emergence of a pattern of regularly spaced of humps of similar species could not so easily be obtained analytically from the competition equations, and was only found by computer simulations 40 years later [13]. The fundamental mathematical mechanism behind such spontaneous pattern formation was in fact uncovered by the mathematician Alan Turing in an 1952 article on the question how an embryo can develop from symmetrical spherical blastula stage into a hydra with tentacles or another animal [16]. The same Turing was also one of the minds behind the development of the modern computer that (as he foresaw in his 1952 paper) would later facilitate more specific predictions of emergent regular patterns (such as ours) now broadly known as *Turing patterns*.

On the empirical side an important precursor we wish to highlight is a seminal paper by C.S. Holling showing that the distribution of body sizes in various groups of animals and habitats is conspicuously ‘lumpy’ [24]. Although his *textural discontinuity hypothesis* suggesting that gaps in distributions of species sizes would reflect gaps in the scales of ecosystem processes and structures has remained controversial [31] Holling’s observations inspired broad follow-up work [32]. Equally controversial, yet ground-breaking was the *neutral theory of biodiversity* by tropical ecologist Stephen Hubbell asserting that most species might be able to co-exist simply because they are essentially equivalent [9]. These theories are not mutually exclusive. Although textural properties of ecosystems would not by themselves explain a regular spacing of body sizes [15], they may still favour certain body sizes that serve as anchor points for an otherwise self-organized regular pattern of body sizes [13]. Similarly, neutrality [9] may seem at odds with niche separation, but in fact ‘near neutrality’ may an important factor facilitating co-existence within a functional niche [13].

Sticking to the metaphor of standing on the shoulders of giants, we would argue that different shoulders supported isolated building blocks: competition theory, Turing instability, lumpiness of species sizes and neutrality of species are elements that now turn out fit seamlessly together. The striking regularity of the patterns we reveal in the size distributions of the world’s 4168 diving beetles, and the consistency across scales and habitats implies a strong case for the view that Turing instability has driven the immense numbers of species on earth to self-organize evolutionary into a surprisingly limited number of functional groups.

### Implications for Ecological Resilience

Obviously, functional redundancy does not imply that species are otherwise identical. Rather, they will typically differ in their response to a range of environmental factors and this response diversity will promote resilience of the group as a whole to various kinds of shocks and fluctuations [33]. Although the species richness and the strong link between body size and niche make these beetles an ideal group for the kind of analysis we performed, there is no reason to assume that similar evolutionary self-organization would not occur in other organisms. Indeed, patterns in organisms ranging from phytoplankton to birds and fossil branchiopods are consistent with this idea [15,19] and recent work on Cichlids convincingly shows how co-occurring species from different phylogenetic origin have evolved to become almost indistinguishable morphologically and ecologically [27].

In conclusion, we suggest that evolution is a generator not only of functional complementarity but also of functional redundancy. While functional complementarity promotes the magnitude of ecosystem processes, redundancy promotes resilience of such ecosystem processes through the insurance effect of biodiversity [33,34]. This insurance effect is due to the fact that species that are near-neutral when it comes to their functional role (e.g. their niche in terms of the food they eat), will typically still differ in their response to various stressors [33]. Such response diversity may include sensitivity to specific parasites and diseases [35]. As a result the resilience of a functional role should be expected to increase with the number of species in a near-neutral group. A corollary is that such resilience from functional redundancy will be much rarer in larger animals, simply because species richness decreases with body size in the animal kingdom. It is therefore no surprise that the loss of large species can give rise to substantial functional change in ecosystems [36]. While redundancy may be the rule in smaller creatures, the functional uniqueness of larger ones could imply that they are often the Achilles heel for ecological functioning.

## Supporting Information

S1 File Description of the model used to produce Fig 1. (PDF)

S2 File Mode analyses methods and tables. (PDF)

## References

[1] Hutchinson GE (1959) Homage to Santa-Rosalia or why are there so many kinds of animals? American Naturalist 93: 145–159.

[2] MacArthur RH, Levins R (1967) Limiting similarity, convergence and divergence of coexisting species. American Naturalist 101: 377–385.

[3] Paine RT (1966) Food web complexity and species diversity. American Naturalist 100: 65–75.

[4] Huisman J, Weissing FJ (1999) Biodiversity of plankton by species oscillations and chaos. Nature 402: 407–410.

[5] Sommer U (1984) The paradox of the plankton: Fluctuations of phosphorus availability maintain diversity of phytoplankton in flow-through cultures. Limnology and Oceanography 29: 633–636.

[6] Hutchinson GE (1961) The paradox of the plankton. American Naturalist 95: 137–145.

[7] Huston MA (1979) A general hypothesis of species diversity. American Naturalist 113: 81–101.

[8] Connell JH (1978) Diversity in tropical rain forests and coral reefs—high diversity of trees and corals is maintained only in a non-equilibrium state. Science 199: 1302–1310. 17840770 10.1126/science.199.4335.1302

[9] Hubbell SP (2001) The Unified Neutral Theory of Biodiversity and Biography; Levin SA, Horn HS, editors. Princeton: Princeton University Press. 375 p.

[10] Bell G (2000) The distribution of abundance in neutral communities. American Naturalist 155: 606–617. 10777433 10.1086/303345

[11] Yu DW, Terborgh JW, Potts MD (1998) Can high tree species richness be explained by Hubbell's null model? Ecology Letters 1: 193–199.

[12] Ricklefs RE (2003) A comment on Hubbell's zero-sum ecological drift model. Oikos 100: 185–192.

[13] Scheffer M, Van Nes EH (2006) Self-organized similarity, the evolutionary emergence of groups of similar species. Proceedings of the National Academy of Science of the United States of America 103: 6230–6235. 10.1073/pnas.0508024103 PMC1458860 16585519

[14] Cadotte MW (2007) Concurrent niche and neutral processes in the competition-colonization model of species coexistence. Proceedings of the Royal Society B: Biological Sciences 274: 2739–2744. 17711831 10.1098/rspb.2007.0925 PMC2279221

[15] Vergnon R, Dulvy NK, Freckleton RP (2009) Niches versus neutrality: Uncovering the drivers of diversity in a species-rich community. Ecology Letters 12: 1079–1090. 10.1111/j.1461-0248.2009.01364.x 19747181

[16] Turing AM (1952) The chemical basis of morphogenesis. Philosophical Transactions of the Royal Society of London Series B-Biological Sciences 237: 37–72. 10.1098/rstb.2014.0218 PMC4360114 25750229

[17] Fort H, Scheffer M, Van Nes EH (2010) The clumping transition in niche competition: A robust critical phenomenon. Journal of Statistical Mechanics: Theory and Experiment 2010: P05005.

[18] Segura AM, Calliari D, Kruk C, Conde D, Bonilla S, Fort H. (2011) Emergent neutrality drives phytoplankton species coexistence. Proceedings of the Royal Society B: Biological Sciences 278: 2355–2361. 10.1098/rspb.2010.2464 21177680 PMC3119015

[19] Vergnon R, Van Nes EH, Scheffer M (2012) Emergent neutrality leads to multimodal species abundance distributions. Nature Communications 3: 663 10.1038/ncomms1663 22314359

[20] Segura AM, Kruk C, Calliari D, García-Rodriguez F, Conde D, Widdicombe CE et al (2013) Competition drives clumpy species coexistence in estuarine phytoplankton. Scientific Reports 3 10.1038/srep01037 23301158 PMC3539148

[21] Loreau M (2004) Does functional redundancy exist? Oikos 104: 606–611.

[22] Holt RD (2006) Emergent neutrality. Trends in Ecology and Evolution 21: 531–533. 16901580 10.1016/j.tree.2006.08.003

[23] Nee S, Colegrave N (2006) Ecology—Paradox of the clumps. Nature 441: 417–418. 16724048 10.1038/441417a

[24] Holling CS (1992) Cross-scale morphology, geometry, and dynamics of ecosystems. Ecological Monographs 62: 447–502.

[25] Nilsson AN, editor (1996) Aquatic Insects of North Europe A Taxonomic Handbook. Volume 1 Stenstrup: Apollo Books. 274 p.

[26] Vergnon R, Leijs R, van Nes EH, Scheffer M (2013) Repeated parallel evolution reveals limiting similarity in subterranean diving beetles. American Naturalist 182: 67–75. 10.1086/670589 23778227

[27] Muschick M, Indermaur A, Salzburger W (2012) Convergent evolution within an adaptive radiation of cichlid fishes. Current Biology 22: 2362–2368. 10.1016/j.cub.2012.10.048 23159601

[28] Leys R, Watts CHS, Cooper SJB, Humphreys WF (2003) Evolution of subterranean diving beetles (Coleoptera: Dytiscidae: Hydroporini, Bidessini) in the arid zone of Australia. Evolution 57: 2819–2834. 14761060 10.1111/j.0014-3820.2003.tb01523.x

[29] Leijs R, van Nes EH, Watts CH, Cooper SJB, Humphreys WF, Hogendoorn K (2012) Evolution of blind beetles in isolated aquifers: A test of alternative modes of speciation. PLoS ONE 7: e34260 10.1371/journal.pone.0034260 22479581 PMC3316697

[30] Schwilk DW, Ackerly DD (2005) Limiting similarity and functional diversity along environmental gradients. Ecology Letters 8: 272–281.

[31] Baskin Y (1997) The rules of life in a lumpy world. Science 275: 311–311.

[32] Allen CR, Holling CS, editors (2008) Discontinuities in Ecosystems and Other Complex Systems Columbia and Princeton: University Presses of California. 272 p.

[33] Elmqvist T, Folke C, Nystrom M, Peterson G, Bengtsson J, Walker B, et al (2003) Response diversity, ecosystem change, and resilience. Frontiers in Ecology and the Environment 1: 488–494.

[34] Loreau M (2000) Biodiversity and ecosystem functioning: recent theoretical advances. Oikos 91: 3–17.

[35] Schnitzer SA, Klironomos JN, HilleRisLambers J, Kinkel LL, Reich PB, Xiao K, et al (2011) Soil microbes drive the classic plant diversity-productivity pattern. Ecology 92: 296–303. 21618909 10.1890/10-0773.1

[36] Estes JA, Terborgh J, Brashares JS, Power ME, Berger J, Bond WJ, et al (2011) Trophic downgrading of planet earth. Science 333: 301–306. 10.1126/science.1205106 21764740

