# Supplementary material for: The Evolution of Functionally Redundant Species; Evidence from Beetles
Source: PLoS One. 2015 Oct 8;10(10):e0137974. doi: 10.1371/journal.pone.0137974 (PMC4598105; doi:10.1371/journal.pone.0137974)
Supplement: S1 File — (PDF) [file pone.0137974.s001.pdf]

Supporting online material

Self-Organized Functional Redundancy in Beetles

Marten Scheffer[1], Remi Vergnon[1], Egbert H. van Nes[1], Jan G.M. Cuppen[1], Edwin T.H.M. Peeters[1], Remko Leijs[2,3,4] and Anders N. Nilsson [5]

S1 Model description

To generate the species size distribution presented in Fig. 1 of the main text we used the model described in the original theory paper of self-organized similarity [13]. The only difference is that we now use a finite niche axis (the original model mostly used a circular niche axis to exclude edge effects), and assume extinction probabilities to be larger at the edges of the niche axis than in the centre. Here is a summary of the model.

$$\frac{dN_i}{dt} = r\,N_i\left(K_i - \sum_j \alpha_{i,j}\,N_j\right)/K_i - g\frac{N_i^2}{N_i^2 + H^2} \qquad (1)$$

i = 1, 2, ..n; $\alpha_{i,i} = 1$

Where N_i is the density of the species i, r is the maximum per capita growth rate, K_i is the carrying capacity of species i, and $\alpha_{i,j}$ is the competition coefficient scaling the effect of species j on species i. The effect of density dependent regulation by parasites, disease or predators is included by the last term in the equation assuming losses to increase (up to a maximum g) when population density exceeds a threshold (H).

To compute competition coefficients that allow us to mimic competition of species for resources along a niche axis (Eq. 1) we characterize the width of the niche by normal distributions on the niche axis (L):

$$P_i(L) = \frac{1}{\sigma\sqrt{2\pi}} e^{-(L-\mu_i)^2/(2\sigma^2)} \qquad (2)$$

We assume that competition intensity between species i and species j is related to niche overlap, and thus to the probability P that individuals of the two species are at the same position on the niche axis, which is the product of both probabilities, and for a finite linear niche axis of length L_{max} becomes:

$$\int_0^{L_{\max}} P_i(L)P_j(L)\,dL \qquad (3)$$

and the competition coefficients are computed as:

$$\alpha_{i,j} = \frac{\int_0^{L_{\max}} P_i(L)P_j(L)\,dL}{\int_0^{L_{\max}} P_i(L)^2\,dL} = e^{-\frac{(\mu_j-\mu_i)^2}{4\sigma^2}} \frac{erf\left(\frac{2L_{\max}-\mu_i-\mu_j}{2\sigma}\right)+erf\left(\frac{\mu_i+\mu_j}{2\sigma}\right)}{erf\left(\frac{L_{\max}-\mu_i}{\sigma}\right)+erf\left(\frac{\mu_i}{\sigma}\right)} \quad (4)$$

Time in the model is scaled in units of r (so r=1) and the carrying capacity K is set default to 10. We used a fourth order Runga-Kutta solver as implemented in MATLAB. For our simulations five hundred species were assigned randomly (following a uniform distribution) to a certain position (μ_i) on the niche axis, each with the same niche width (standard deviation σ = 0.0475).

To mimic evolution, each species iterates its position on the niche axis each 1000 time steps to increase its 'fitness'. The 'fitness' of the species is defined as the inverse of the carrying capacity that an invader would need to invade successfully which can be computed from the condition for positive growth at low initial density [2]:

$$K_i > \sum_j \alpha_{i,j}\ N_j$$

The evolutionary step-size is set to 0.01 niche units and the species move on the niche axis in the direction where their fitness increases. In addition we allow for a stochastic extinction 500 time steps after each evolutionary time step where extinction probabilities, following cosine function that is scaled to have an extinction probability of 0 in the centre and P_{max} at the edges of the niche axis (at L_i=0 and L_i=L_{max}):

$$P_{ext,i} = \left(1-(0.5+0.5\cos\left(\left(\frac{L_i+0.5L_{\max}}{L_{\max}}\right)2\pi\right)\right)P_{\max}$$

To keep the total number of species constant we re-initiate species (the same number of species that went extinct) on random position on the niche axis.

S2 Analysis of multimodality

To detect multiple modes in the frequency distributions of body lengths we fitted Gaussian distributions through latent class analysis using the ‘gmdistribution’ function from the MATLAB statistics toolbox (MATLAB version R2011a). The use of such ‘finite mixture models’ is a good solution in situations where observations may come from different components - in our case different modes along the abundance distributions - and where it is unknown which group each observation belongs to. Latent Class analysis consists of fitting a model with a certain number of components (here Gaussian distributions) by maximizing the likelihood of data using the Expectation Maximization (EM) algorithm. As the EM algorithm is an iterative method that might find local (suboptimal) minima, we fitted each model 20 times with different initial guesses. To find the optimal number of components, we fit series of finite mixture models with increasing

number of components (1-10), determining which number of components best explains observed data based on the minimum Akaike Information Criterion (AIC, see tables below).

A general background on this class of statistics can be found for instance in: McLachlan, G., and D. Peel, Finite Mixture Models, John Wiley & Sons, New York, 2000. The kernel density estimate curves (Gaussian kernel, band width 0.06) we give represent a kind of weighted running averages to provide a visual check of the number of modes.

Table 1. Akaike Information Criterion values for models with different number of modes (1-10) for each region. The optimal numbers of clusters (with the lowest AIC value) are represented in bold, the other values are the differences with the optimum AF= Afrotropic, PL = Palearctic (incl all of China), NT = Neotropic (South and Central America); OR= Orientalis (also Indomalaya, roughly India, S.E. Asia); AU = Australasia; NA = Nearctic (US and Canada); North= NA+PL; South=AF+NT+OR+AU, and Total = all data.

N clusters	AF	PL	NT	OR	AU	NA	North	South	Total
1	+375	+195	+161	+190	+60	+77	+257	+631	+627
2	+146	+127	+56	+80	+21	+57	+160	+295	+279
3	+67	+96	+15	+17	+14	+33	+106	+100	+90
4	+7	+31	+2	+1	+0	+9	+30	+10	+3
5	**1995**	+11	+1	**983**	**690**	**939**	+13	**5365**	**7757**
6	+6	+6	**1546**	+10	+3	+0	+14	+2	+6
7	+9	**1816**	+12	+16	+6	+4	+1	+3	+6
8	+6	+1	*	+22	+10	+10	**2660**	+8	+13
9	+5	+7	*	+19	+12	+12	+5	+15	+19
10	+11	+10	*	+18	+10	+22	+11	+14	+19

*= failed to converge

Table 2. Positions of the modes for each of the clusters for the optimal model according to the Akaike Information Criterion for each region. AF= Afrotropic, PL = Palearctic (incl all of China), NT = Neotropic (South and Central America); OR= Orientalis (also Indomalaya, roughly India, S.E. Asia); AU = Australasia; NA = Nearctic (US and Canada); North= NA+PL; South=AF+NT+OR+AU, and Total = all data.

Cluster No.	AF	PL	NT	OR	AU	NA	North	South	Total
1	0.833	0.669	0.615	0.559	1.01	0.626	0.673	0.728	0.694
2	1.4	0.942	0.997	1.32	1.5	1.32	0.952	1.44	1.35
3	1.76	1.21	1.37	1.59	1.85	2.07	1.19	1.85	1.63
4	2.59	1.5	1.8	2.53	2.46	2.69	1.48	2.49	2.53
5	3.46	2.1	2.38	3.3	3.25	3.36	1.79	3.35	3.36
6		2.69	3.28				2.09		
7		3.33					2.69		
8							3.35		
