# Supplementary material for: The Evolution of Functionally Redundant Species; Evidence from Beetles
Source: PLoS One. 2015 Oct 8;10(10):e0137974. doi: 10.1371/journal.pone.0137974 (PMC4598105; doi:10.1371/journal.pone.0137974)
Supplement: S2 File — (PDF) [file pone.0137974.s002.pdf]

Supporting online material

Self-Organized Functional Redundancy in Beetles

Marten Scheffer[1], Remi Vergnon[1], Egbert H. van Nes[1], Jan G.M. Cuppen[1], Edwin T.H.M. Peeters[1], Remko Leijs[2,3,4] and Anders N. Nilsson [5]

Table S1. Akaike Information Criterion values for models with different number of modes (1-10) for each region. The optimal numbers of clusters (with the lowest AIC value) are represented in bold. AF= Afrotropic, PL = Palearctic (incl all of China), NT = Neotropic (South and Central America); OR= Orientalis (also Indomalaya, roughly India, S.E. Asia); AU = Australasia; NA = Nearctic (US and Canada); North= NA+PL; South=AF+NT+OR+AU, and Total = all data.

N clusters	AF	PL	NT	OR	AU	NA	North	South	Total
1	2370	2011	1707	1173	750	1016	2917	5996	8384
2	2141	1943	1602	1063	711	996	2820	5660	8036
3	2062	1912	1561	1000	704	972	2766	5465	7847
4	2002	1847	1548	984	690	948	2690	5375	7760
5	**1995**	1827	1547	**983**	**690**	**939**	2673	**5365**	**7757**
6	2001	1822	**1546**	993	693	939	2674	5367	7763
7	2004	**1816**	1558	999	696	943	2661	5368	7763
8	2001	1817	*	1005	700	949	**2660**	5373	7770
9	2000	1823	*	1002	702	951	2665	5380	7776
10	2006	1826	*	1001	700	961	2671	5379	7776

*= failed to converge

Table S2. Positions of the modes for each of the clusters for the optimal model according to the Akaike Information Criterion for each region. AF= Afrotropic, PL = Palearctic (incl all of China), NT = Neotropic (South and Central America); OR= Orientalis (also Indomalaya, roughly India, S.E. Asia); AU = Australasia; NA = Nearctic (US and Canada); North= NA+PL; South=AF+NT+OR+AU, and Total = all data.

Cluster No.	AF	PL	NT	OR	AU	NA	North	South	Total
1	0.833	0.669	0.615	0.559	1.01	0.626	0.673	0.728	0.694
2	1.4	0.942	0.997	1.32	1.5	1.32	0.952	1.44	1.35
3	1.76	1.21	1.37	1.59	1.85	2.07	1.19	1.85	1.63
4	2.59	1.5	1.8	2.53	2.46	2.69	1.48	2.49	2.53
5	3.46	2.1	2.38	3.3	3.25	3.36	1.79	3.35	3.36
6		2.69	3.28				2.09		
7		3.33					2.69		
8							3.35		
